# A nurse-led health coaching intervention for stroke survivors and their family caregivers in hospital to home transition care in Chongqing, China: a study protocol for a randomized controlled trial

**DOI:** 10.1186/s13063-020-4156-z

**Published:** 2020-03-04

**Authors:** Shuanglan Lin, Lily Dongxia Xiao, Diane Chamberlain

**Affiliations:** College of Nursing and Health Sciences, Flinders University, GPO Box 2100, Adelaide, SA 5001 Australia

**Keywords:** Stroke, Stroke survivors, Caregivers, Transitional care, Health coaching, Self-efficacy, Quality of life, Randomized controlled trial

## Abstract

**Background:**

Hospital to home transition care is a most stressful period for stroke survivors and their caregivers to learn self-management of stroke-related health conditions and to engage in rehabilitation. Health coaching has been identified as a strategy to enhance self-management of poststroke care at home. However, interventions in this field that are informed by a health coaching framework are scarce. This study will address a gap in research by testing the hypothesis that a nurse-led health coaching intervention can improve health outcomes for stroke survivors and their family caregivers in hospital to home transition care.

**Methods:**

This is a single-blind, two-arm parallel randomized controlled trial of a nurse-led health coaching program versus routine care situated in two tertiary hospitals in Chongqing, China. Stroke survivors and their primary family caregivers will be recruited together as “participant dyads”, and the estimated sample size is 140 (70 in each group). The intervention includes a 12-week nurse-led health coaching program in hospital to home transition care commencing at discharge from the hospital. The primary outcome is changes in self-efficacy of stroke survivors at 12 weeks from the baseline. The secondary outcomes are changes in stroke survivors’ and quality of life, functional ability, stroke-related knowledge, the number of adverse events, and unplanned hospital admissions, and caregivers’ self-efficacy and caregiver-related burden at 12 weeks from the baseline. The outcomes will be measured at 12 weeks and 24 weeks from the baseline.

**Discussion:**

This study will examine the effect of nurse-led health coaching on hospital to home transition care for stroke survivors and their caregivers. It is anticipated that findings from this trial will provide research evidence to inform policy, and resource and practice development to improve hospital to home transition care for stroke survivors and their caregivers.

**Trial registration:**

The Australian New Zealand Clinical Trials Registry (ANZCTR): ACTRN12619000321145. Registered on 1 March 2019.

## Background

Stroke contributed to 5.9 million deaths and 102 million severe adult disabilities globally in 2010 [1]. Up to 70% of stroke deaths and 78% of stroke associated disability-adjusted life-years (DALYs) lost occur in low- and middle-income countries (LMICs) including China [1]. In China, health loss is mainly caused by stroke, and the morbidity and mortality rates of stroke show an increase over the past 20 years [2]. It is estimated that there are two million new-onset stroke survivors each year in China, making this the second highest incidence of stroke in the world [3]. Of stroke survivors, 75% lose the ability to work, 40% have severe disability and 25%–33% suffer a second-time stroke [4]. Hospital to home transition is viewed as the most challenging period of time for stroke survivors and their family caregivers to adapt self-care at home while engaging in rehabilitation to gain full potential of functional ability and social integration [3, 5, 6]. However, transition care services are largely underdeveloped in most LMICs including China [7, 8]. About 80% of stroke survivors have to discharge to home for later physical and functional rehabilitation due to deficient rehabilitation centers and nursing homes in China [9–11]. Family caregivers play a crucial role in assisting stroke survivors in self-care and rehabilitation during hospital to home transition [12, 13]. However, they usually received little preparation for their caregivers’ role. It is reported that a lack of support for caregivers is associated with physical and psychological stress, caregiving burden and a low level of quality of life [13–16]. Transition care interventions therefore need to consider support for caregivers. This study addresses a gap in research by implementing and evaluating an evidence-based health coaching intervention for stroke survivors and their family caregivers in hospital to home transition in Chongqing, China.

Maladaptation of stroke survivors to self-care at home after hospital discharge is associated with complications, adverse events, high readmission rates to hospital, delayed recovery, psychological and emotional stress, social isolation and a low level of quality of life [5, 6]. Therefore, hospital to home transition care for this patient population has become a priority care area to optimize care outcomes and relieve burdens on the healthcare system [17]. Health coaching is an effective strategy to enhance transition care for stroke survivors and their caregivers. In this study, we adapt the definition of health coaching by Wolever and colleagues described as “a patient-centered approach wherein patients at least partially determine their goals, use self-discovery or active learning processes together with content education to work toward their goals, and self-monitor behaviors to increase accountability, all within the context of an interpersonal relationship with a coach” [18]. Although considerable evidence supports the effectiveness of health coaching on quality of life and activities of daily living in other chronic diseases [19–23], findings from the mentioned studies indicate that the effect on stroke survivor’s health outcomes varies.

The aim of this study is to determine the effectiveness of nurse-led health coaching for stroke survivors and primary caregivers in hospital to home transition care in Chongqing, China. We hypothesis that stroke survivors in the health coaching intervention group will demonstrate better health outcomes at 12 weeks from the baseline compared to the usual care group.

## Methods/design

### Study design

**Fig. 1:**
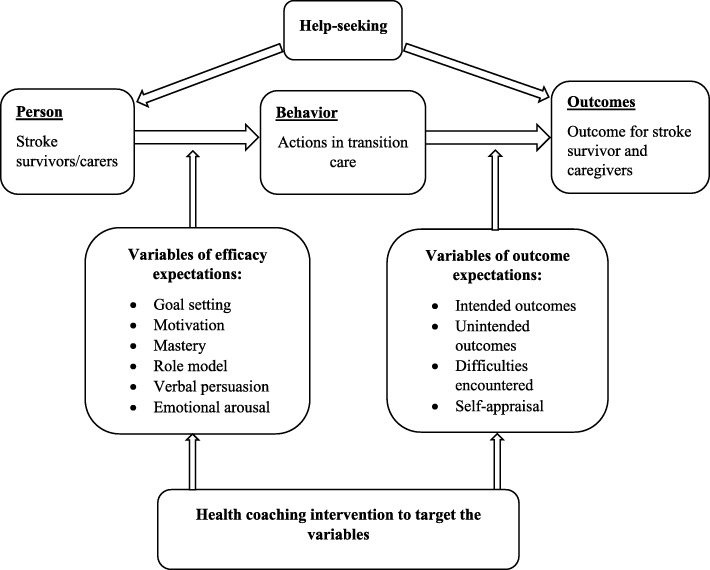
Flow chart of study design

This is a single-blind, two-arm parallel randomized controlled trial of a nurse-led health coaching program for stroke survivors versus routine care situated in the First Affiliated Hospital and the Third Affiliated Hospital of Army Medical University in Chongqing, China (Fig. 1). These hospitals are chosen as they are nearly identical regarding patient population and staff profiles. They serve for the same civilian population in a large metropolitan area and are administered by a similar governance body, policies, standards; and have same staffing levels and education and training for staff. The intervention will last 12 weeks. Data collection will be at three time points: the baseline (after the consent form is signed), immediately after the intervention (at 12 weeks), and 24 weeks from the baseline. Stroke survivor-caregiver dyads will participate in the study and will be recruited from the hospitals mentioned above. Four stroke wards from these tertiary hospitals (two from each hospital) will participate in the study. The care services provided to stroke patients in these wards are similar. The nurse-led health coaching program will be conducted at stage 1 in the hospital and stage 2 in the community after discharge from hospital. The trial follows the Recommendations for Intervention Trials (SPIRIT) that is presented in Additional file 1.

### Ethical approvals and consent to participant

The protocol was approved by the Southern Adelaide Clinical Human Research Ethics Committee (Approval Number: 230.18) and the Institutional Review Boards of the First Affiliated Hospital and the Third Affiliated Hospital of the Army Medical University, China. Informed consent will be obtained from all participants. Any information provided by participants will be collected in a de-identifiable form. Each participant and participating health facility will be given a unique numerical code to represent them on data collection forms used throughout the project. Any information provided to the study will be treated in strict confidence, and none of the participants will be individually identifiable in research reports. All paper-based questionnaire surveys and patients and caregivers files will be locked in a safe cabinet in the research office and only the co-investigator will have access to it.

### Eligibility: Inclusion criteria and exclusion criteria

#### Inclusion criteria and exclusion criteria for stroke survivors

Participants will meet all of these inclusion criteria: (1) aged 18 or over; (2) first-time stroke survivors (ischemic or haemorrhagic stroke); (3) will be discharged to home-care setting within a week; (4) have no cognitive impairment (Montreal cognitive assessment score ≥ 23) [24]; (5) not participating in other clinical trials at the same time; (6) can speak Chinese without aphasic; and (7) willing to participate in this study.

Participants will be excluded if they have (1) comorbidities such as severe circulation, digestive, immune, blood and endocrine systems disorders; (2) existing cerebrovascular disease with impaired limb function; (3) mental illness (such as Panic disorder, PTSD, Psychosis, Schizoaffective disorder, Schizophrenia); or (4) existing cognitive impairment (depression and delirium) and disabilities prior to stroke .

#### Inclusion criteria and exclusion criteria for caregivers

Caregivers will meet all these inclusion criteria: (1) aged 18 or over; and (2) be the primary caregiver. Caregivers will be excluded if they (1) are under 18 years old; or (2) have mental or cognitive disabilities.

#### Inclusion criteria for health coaches

Health coaches will be trained intervention nurses from each participating ward of the two hospitals, and they will conduct all interventions in this study. The eligibility criteria for health coaches are (1) a registered nurse; (2) at least a bachelor’s degree in nursing; and (3) at least 5-years work experience in the care of stroke survivors.

### Randomization

We will allocate eligible participant dyads to the intervention group or the control group using simple randomization, with an equal allocation ratio of 1:1, using IBM SPSS Statistics version 25 random sampling and allocation methods. A research associate who is not involved in recruitment, intervention allocation or outcome assessment will generate the random numbers. Allocation concealment will be ensured by the research associate putting the group allocation into sequentially numbered, opaque, sealed envelopes to inform the intervention nurse after baseline data collection for each dyad.

### Evaluator blinding

Participant dyads and intervention nurses will not be blinded due to the nature of the intervention. However, the researcher, who will collect the outcome assessments and undertake data entry and analysis, will remain blinded to treatment allocations. Intervention nurses, who will conduct the telephone and outpatient clinic follow-ups, will not be blinded as they will need to update participant information and maintain the intervention and service.

### Description of the interventions

Interventions that will be implemented in this study are based on effective health coaching components identified in a systematic review and meta-analysis conducted by the research team prior to this RCT study and other systematic reviews and studies on health coaching in chronic condition management [20, 22, 25, 26]. A two-stage intervention for the intervention group will be conducted as described in the following.

### Stage I: Interventions while in hospital

An intervention nurse will provide participant dyads with individualized health coaching within one week prior to discharge from hospital.

Individualized health coaching: intervention nurses will provide participant dyads with two face-to-face individualized health coaching sessions (20–30 min each). All health coaching sessions will be based on the “Health Coaching Intervention Program Guide” (Table 1) to standardize interventions in the study.

**Table 1:** The guide for health coaching intervention program

Health coaching session	Items	Topics
Three days prior to discharge	Self-care skills	To demonstrate the skills of personal hygiene, dressing, eating, maintaining continence and transferring, and how to record health coaching diary for self-monitoring.
	Creating a safe home environment	To assess the home environment according to the stroke survivors’ needs. To demonstrate and discuss how to create a safe home environment. Examples will be provided in relation to the increase of the lighting in the living room, bedroom and toilet. Minimizing the furniture and provide the patient with an accessible area. Creating floor with non-slip, no steps, and to install handrails on the walls.
	Functional ability rehabilitation and exercise plan	Based on the plan provided by medical specialist, the coach will demonstrate how to perform the recommended activities, provide opportunity for stroke survivors to perform these activities and gain feedback from the coach. The training also includes, but not limited to: • Maintain the correct posture and correct the abnormal pattern. • Maintain the functional position of the paralysed limb to prevent malformation. • Active and passive function training on hemiplegic side. • Guide the transfer training, swallowing function training.
Two days prior to discharge	Medication management	• Give health education about medication use, monitoring side effects and medication adherence. • Demonstrate how to record the medication using Health Coaching Diary.
	Complication prevention	• To assess the complication prevention according to the stroke survivors’ condition. • To demonstrate how to prevent, identify and manage stroke-related complications(second stroke, bedsore, falls and urinary tract infection).

Sources: 1. Stroke Foundation. National Stroke Audit – Rehabilitation Services Report 2016. Melbourne, Australia. 2. National Stroke Foundation. Clinical Guidelines for Stroke Management 2010. Melbourne, Australia. 3. https://strokefoundation.org.au/

Outline of health coaching activities:

1. Participant dyads will establish their transition care goals and develop personal action plans.
2. An intervention nurse will demonstrate self-care and other skills required by participant dyads when providing Activities of Daily Living (ADLs) and Instrumental Activities of Daily Living (IADL) and participating in social engagements.
3. Participant dyads will discuss a safe home environment including safe toileting, walking and sleeping. Home environment modifications will be undertaken prior to discharge when needed.
4. An individualized functional recovery and physical exercise plan will be developed based on the recommendation from the stroke survivor’s medical specialist. An intervention nurse will discuss the plan and demonstrate skills when appropriate. The functional recovery and physical exercise plan will be recorded in the “Stroke Health Coaching Diary” (Additional file 2).
5. Stroke survivors will be given an individualized treatment and rehabilitation plan provided by medical specialists, have opportunities to discuss and clarify the plan and receive medication education from an intervention nurse. The treatment and rehabilitation plan will be recorded in the “Stroke Health Coaching Diary”.
6. An intervention nurse will demonstrate how to prevent, identify and manage stroke-related complications.

### Stage II: Follow-up interventions in the first 12 weeks after discharge to home

Intervention nurses will provide participant dyads with phone support and face-to-face meetings in outpatient clinics over a 12-week period as follows:

1. Weekly telephone support: Participant dyads will receive a weekly telephone call at home from an intervention nurse. Participants will be encouraged to discuss concerns and problems arising from transition care. They will also be provided with resources and strategies to resolve problems. An intervention nurse will motivate participants to monitor their care activities and outcomes towards their transition care goals. Each phone call will last about 10–15 min. Participants can also seek additional phone help from an intervention nurse when necessary.
2. Bi-weekly face-to-face coaching activities at an outpatient department: Stroke survivors will engage in rehabilitation or have an appointment with specialists at the outpatient clinic bi-weekly. An intervention nurse will use this opportunity to meet with participant dyads. Participant dyads will be asked to bring their coaching diary to this meeting to lead discussion about any concerns and problems they have encountered. An intervention nurse will coach participants to develop problem-solving skills based on their needs, introduce resources to them, reinforce self-care knowledge and skills, and motivate them to self-monitor progress and outcomes towards care goals.

### Usual care

Participants in the usual care group will receive the usual transitional discharge plan that includes verbal stroke-related education and a follow-up telephone call at the end of the first and the fourth week after discharge to home. For usual care, other registered nurses, who are not intervention nurses, will conduct the discharge plan and follow-up.

### Outcomes measures

**Fig. 2:**
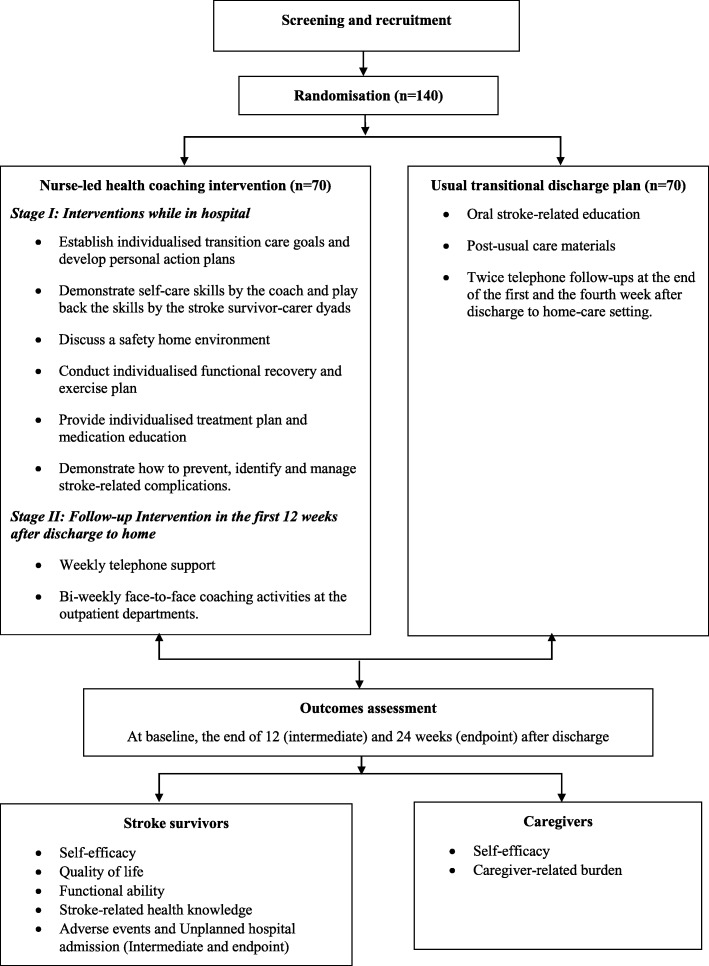
SPIRIT figure: general procedure of enrolment, intervention and assessment

Outcome measures are based on hypotheses of this RCT. The effects of the intervention will be measured at baseline, 12 weeks and 24 weeks. The general procedure of data collection and assessments is presented in Fig. 2 (the SPIRIT figure).

### Outcomes of stroke survivors

#### Primary outcomes

1.1. Self-efficacy: This outcome will be measured using the Stroke Self-efficacy Questionnaire (SSEQ). The SSEQ consists of 13 items to test self-efficacy in two dimensions: activity domain (7 items) and self-management (5 items) [27]. It has been translated into Chinese and proven to be reliable and valid for the Chinese population with the Cronbach’s α 0.972, and a test-retest reliability coefficient of 0.806 [28].

#### Secondary outcomes

2.1 Quality of life: This outcome will be measured using the short version of the Stroke-Specific Quality of Life Scale (SSQoL-12). The SSQoL-12 is a self-assessment scale comprising physical domain (6 items) and psychosocial domain (6 items) [29]. The SSQoL-12 has good criterion validity and internal consistency (Cronbach’s α 0.77–0.89).
2.2 Functional ability: This outcome will be measured using (1) the Chinese version of the Barthel Index (BI) for ADL, (2) the Multilevel Lawton Instrumental Activities of Daily Living (Lawton IADL), and (3) the Social Support Rating Scale (SSRS). The BI comprises 10 items and is a valid tool to evaluate the ADL of stroke survivors [30, 31]. It has been translated into Chinese with acceptable internal consistency (Cronbach’s α of 0.916) [32]. The Lawton IADL is an appropriate instrument with eight categories to assess independent living skills [33]. It has been used and proven in the Chinese population with a satisfactory Cronbach’s alpha of 0.739 [34]. The SSRS was developed in China and is widely used with high validity (Cronbach’s alpha 0.83 to 0.90) and reliability (test–retest reliability 0.92) [35, 36].
2.3 Stroke-related health knowledge: This outcome will be measured using the Chinese Stroke Prevention Knowledge Questionnaire (SPKQ). The SPKQ comprises 25 items using close-ended questions to assess patients’ stroke-related knowledge. It has been used in the Chinese population with a validity index of 0.89 and Cronbach’s α of 0.86 [37].
2.4 The number of deaths, adverse events and unplanned hospital admissions: The adverse events will be limited to secondary strokes, falls, ulcers and urinary tract infections to reflect the nurse-led intervention to prevent post-stroke associated complications. These outcomes will be recorded by participants on the “Adverse Events Form” and verified by an intervention nurse.

### Outcomes of Caregivers

1. Self-efficacy: This outcome will be measured using the Generalized Self-Efficacy Scale (GSES). It contains 10 items to measure people at risk for coping deficiencies. With its highly validity, the GSES has been widely used around the world. It has been used in the Chinese population with a Cronbach’s α of 0.89. Higher scores indicate better self-efficacy [38, 39].
2. Caregiver-related burden: This outcome will be measured using the Chinese version of the Modified Caregiver Strain Index (CSI). The CSI contains 13 items including financial, physical, psychological, social and personal domains. It is rated on a scale from 0 (No) to 2 (Yes). The total score ranges from 0 to 26 points, with higher scores indicating greater caregiver burden [40]. It was validated in the Chinese population with a Cronbach’s alpha of 0.91 [41].

### Sample size

The sample size is estimated on the basis of the primary outcome, stroke survivors’ self-efficacy, measured by the SSEQ [27]. We generated the estimated effect size of 0.5 based on a 7.5 point between-group change in SSEQ from baseline reported in a similar RCT by Lo et al. [42]. We also estimated a pooled SD of 6.42 based on the 95% confidence interval of the SSEQ score reported in the study. A type I error of alpha 0.05 and a power of 80% were used in the sample size calculation. We expect a 10% dropout rate. Therefore, we will require 70 dyads per treatment group. In total, we will recruit at least 140 participant dyads into the study.

### Recruitment

Stroke survivors and their primary family caregivers will be recruited together as participant dyads from participating wards. A recruitment poster will be displayed at the nursing station of each ward and a secure participant response drop-box placed underneath. Participant dyads who are interested in this project will be asked to fill in a response slip that includes their contact details and place it in the drop-box. Upon receiving the response slip, a research recruiter (the secretary in each ward), who is blinded to group allocation and has no interest in the outcome of the study, will contact participant dyads to confirm their participation. On confirmation of participation, an intervention nurse will check the eligibility of participant dyads, explain the project to them and seek informed consent. An intervention nurse will also allocate each participant dyad to either the intervention group or the usual care group after baseline data collection using the sequentially numbered, opaque, sealed envelopes.

### Data collection methods

Baseline data collection will be conducted at the hospitals through self-administered questionnaires. The researcher (SLL) will prepare a drop box for each ward. Participants will be instructed to place their completed questionnaire in a sealed envelope to maintain confidentiality and to put the envelope into the drop box. The researcher will collect completed baseline questionnaires on a weekly basis. For 12-week and 24-week follow-up data collection, the researcher (SLL) will mail a questionnaire to participants with pre-paid and pre-addressed envelopes. Participants will be instructed to return the completed questionnaire to the researcher by post.

### Data analysis

The intention-to-treat (ITT) approach will be adopted for data analysis. Statistical analysis will be performed using IBM SPSS Statistics version 25 and STATA software version 15. The normality of data will be assessed using normality plots. Percentages will be presented to summarise category data. Means and standard deviation, or medians and IQR, will be presented to describe countinous data with normal distribution and non-normal distribution respectively. Two group comparisons of demographic and clinical characteristics at baseline will be performed using Chi-square tests for category data and independent t-tests or non-parametric Mann-Whitney U-tests for data with normal distribution or non-normal distribution respectively.

The researcher will determine changes in outcome measures at baseline, and 12-week and 24-week follow-ups. To estimate mean changes over time in primary and secondary outcomes, linear mixed effect models for repeated measures will be used to assess differences between the two groups. Fixed effects in the model will include time, group, covariate(s) and the two-way interaction of group and time. The model will also include participant-level random effects to account for correlation between participants’ repeated measures over time. Value and confounders that include age, gender, educational level, insurance type, and number of comorbidities at baseline will be adjusted in this model. A *p* < 0.05 will be regarded as statistically significant for all statistical tests. Estimated mean differences, including 95% confidence intervals and *p*-values, will be reported for (time × group) interaction effects.

### Data monitoring

Data monitoring will be overseen by the Southern Adelaide Clinical Human Research Ethics Committee (Approval Number: 230.18). The Committee is independent from the project team and has no competing interests. Researchers are required to provide annual and/or final report to the Committee regarding data monitoring. Detailed requirements can be accessed via the Committee website [43].

### Quality control

Prior to commencing the project, the researcher (SLL) will introduce the intervention study to each participating ward through a one-hour presentation. The purpose of the presentation is to inform health professionals about the study, gain their support and allow them to ask questions in relation to the study.

Four intervention nurses from participating wards (one from each ward) will participate in two workshops (two hours each) to standardize health coaching interventions throughout the project. The workshops will enable intervention nurses to practice health coaching skills, gain feedback from the researcher and their peers, and ask any questions they may have. The workshops will be provided by the researcher (SLL) who is a qualified academic member of the Nursing College of Chongqing Medical University and a specialist in chronic disease management including the care of stroke survivors. Intervention nurses will also be provided with written “Health Coaching Intervention Program Guidelines” (Table 1).

The researcher (SLL) will recruit 3–5 hospital volunteers (laypersons) for a workshop to test whether they understand the questionnaires and measure the time taken to complete. Each intervention nurse will undertake a pilot coaching activity with workshop volunteers and receive feedback from the researcher. To ensure data quality, double data entry will be conducted by four master’s students who are not involved in this study.

### Intervention fidelity

Standardized training and written guidelines for intervention nurses will enhance adherence to the study protocol. A “Stroke Health Coaching Diary” and a written education booklet to reinforce health coaching activities for stroke survivor-caregiver dyads in the intervention group will be used to help them adhere to the intervention protocol.

### Dealing with contingencies

The first possible contingency in the study is the unforeseen withdrawal of a trained intervention nurse. The strategy to deal with this will be to train another nurse to replace them. The second possible contingency is difficulties in recruiting enough stroke survivor-caregiver dyads to meet the sample size requirement. If required, we will extend the recruitment period to gain enough participants.

## Discussion

**Fig. 3:**
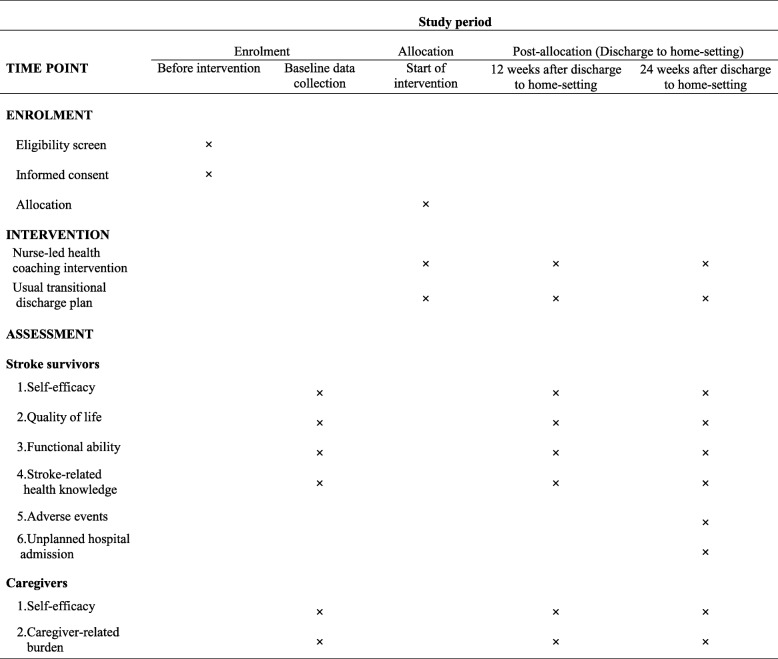
Theoretical framework of the stroke health-coaching program (Adapted from self-efficacy theory)

Self-efficacy theory, a component of social cognitive theory developed by Bandura, focuses on the interrelationships among four concepts that determine a person’s self-efficacy: (1) self-efficacy expectations, (2) behavior, (3) outcome expectations and (4) outcome [44]. Self-efficacy is activated by goal setting through which a person is motivated to take actions (or change behaviors) to achieve their goal. Motivation includes intrinsic motivation (or self-motivation) and extrinsic motivation (being motivated by others, for example, a health coach or a role model). We have adapted self-efficacy theory to health coaching as illustrated in Fig. 3. In our study, stroke survivors and their caregivers will engage in individualized stroke education facilitated by a health coach prior to discharge. This education will equip them with knowledge about health consequences during the hospital to home care transition. A health coach will also work with them to set realistic goals for transition care (activate self-efficacy) and motivate them to achieve their goals (motivated by others). Transitional care goals will be individualized and include aspects of (1) self-care skills, (2) the functional recovery plan (provided by the patient’s medical specialist), (3) medication adherence, (4) maintaining a safe home environment, and (5) prevention of secondary stroke, complications and adverse events.

Health coaching is informed by self-efficacy theory which is widely applied to behavior changes for improved self-management of chronic conditions [44]. Together, the definition and the theory base of health coaching suggest that health coaching interventions should incorporate behavior change techniques to engage participants in the processes of behavior change [45] (Additional file 3). The CALO-RE taxonomy developed by Michie et al. [46], which standardizes behavior change techniques to improve self-efficacy in chronic disease management, is applied to health coaching interventions [45]. The CALO-RE taxonomy targets two areas of behavioral change: (i) barriers to behavior change (or behavior variables), for example, behavioral change goal-setting and motivation to engage in change processes; and (ii) barriers to achieving health outcomes (or outcome variables) including physical and psychosocial well-being outcomes [44, 46].

This study supports the health initiative in China focusing on the prevention and management of stroke. The National Health and Wellness Committee launched this initiative in 2009 and strengthened the stroke comprehensive prevention program in 2016 [47, 48]. These health initiatives aim to reduce the burden on patients, family members, medical resources and society. However, hospital to home transition care was not a focus. Gaps in supporting self-care, rehabilitation, prevention of secondary stroke and reducing caregiver burden still exist in the healthcare system of China compared to high-income countries [48]. It is anticipated that findings from this intervention study will provide research evidence to inform policy initiatives in the care of stroke survivors in hospital to home transition.

It has been argued that the relationship between the health coach and the program participant is crucial to ensure positive health outcomes [18, 49, 50]. Core competencies to help health coaches build support are (1) facilitating participant-centered health coaching activities; (2) helping participants to identify their own goals for change; (3) enabling participant self-discovery of processes to improve self-management; (4) understanding how to help participants be accountable to themselves and self-monitor; (5) knowing how to help participants adapt to the processes of behavior change; and (6) demonstrating interpersonal skills to integrate content information into the change process [18]. We have considered these competencies and embedded them into the workshop training for intervention nurses. Role plays during the training for intervention nurses will also provide opportunities to solve problems in building relationships with participants.

Goal setting in health coaching needs to be individualized, and the goal needs to be achievable for participants [51]. In hospital to home transition care, stroke survivors may experience changes on a weekly basis. Therefore, timely adjustment of transition care goals is imperative. Weekly telephone support, bi-weekly assessments and needs-based phone contact in the post discharge follow-up will enable intervention nurses to work with participants to adjust their care goals when needed.

Physical exercise training for stroke survivors is critical to enable them to optimize poststroke recovery, functional ability and well-being [52, 53]. Moreover, physical exercise typically demands the integration of strength, dosage and duration of repetitive practice according to the patient’s conditions [54]. Although an individualized physical functional recovery and exercise plan will be provided by the medical specialist, in this study intervention nurses will play a key role in motivating stroke survivors to adhere to the plan and encouraging them to provide feedback to their medical specialists to alter the plan when needed.

Fidelity of implementation is an important factor in the intervention’s effectiveness [55]. There is little consensus about key factors affecting intervention fidelity. However, the domains of fidelity, such as study design, training and intervention delivery, have been considered and will be addressed through the following strategies [56]. First, the researchers in this study have adapted the health coaching definition, self-efficacy theory and CALO-RE taxonomy that standardizes health coaching intervention design and behavior change techniques [45, 46]. Second, the training of intervention nurses to standardize health coaching interventions will improve adherence to the intervention protocol. Third, intervention nurses will encourage stroke survivors to record self-care in their health coaching diary to self-monitor processes to achieve set goals. In addition, open-ended questions will be provided at the end of the trial to analyze factors that may affect intervention fidelity.

The protocol has some limitations. First, although all outcomes will be recorded by an independent researcher to minimize the risk of bias, participant dyads and health coaches cannot be blinded due to the nature of the intervention. Second, successful behavior modification is a sustainable long-term process. However, the duration of the intervention and follow-up in this study are 12 and 24 weeks respectively. Therefore, the long-term effectiveness of health coaching in stroke rehabilitation needs to be further explored. In addition, future studies need to consider the measures of cardiovascular events as part of study outcomes when it is appropriate.

## Trial registration

T#he Australian New Zealand Clinical Trials Registry (ANZCTR): ACTRN12619000321145, registered on 1st March 2019.

### Trial status

At the time of the manuscript submission, the participants are being recruited (started on April 8, 2019), and recruitment is expected to be completed on October 10, 2019. The protocol is Version 2.0, dated 20/01/2020.

## Supplementary information

**Additional file 1.** SPIRIT checklist.

**Additional file 2.** Stroke Health Coaching Diary.

**Additional file 3.** The model of consent form.

## Data Availability

The datasets used and/or analyzed during the current study will be available from the corresponding author on reasonable request. The results of the study will be submitted for publications once the data collection and analysis are completed. A summary of global findings will be sent to participants and participating hospitals by email or posting to them with the permission of participants. The outcomes will also be published in the PhD dissemination by the PhD student in the study (SLL).

## Abbreviations

ADL: Activities of Daily Living
BI: Barthel Index
CSI: The Caregiver Strain Index
DALYs: Disability-adjusted life-years
GSES: Generalized Self-Efficacy Scale
IADL: Instrumental Activities of Daily Living
ITT: The intention-to-treat
LMICs: Low- and middle-income countries
PTSD: Post-traumatic stress disorder
QoL: Quality of life
RCT: Randomized controlled trial
SPKQ: Stroke Prevention Knowledge Questionnaire
SSEQ: Stroke Self-efficacy Questionnaire
SSQoL-12: Stroke-Specific Quality of Life Scale
SSRS: Social Support Rating Scale
