# Correction to: A nurse-led health coaching intervention for stroke survivors and their family caregivers in hospital to home transition care in Chongqing, China: a study protocol for a randomized controlled trial

**DOI:** 10.1186/s13063-020-04609-3

**Published:** 2020-08-05

**Authors:** Shuanglan Lin, Lily Dongxia Xiao, Diane Chamberlain

**Affiliations:** College of Nursing and Health Sciences, Flinders University, GPO Box 2100, Adelaide, SA 5001 Australia

**Correction to: Trials 21, 240 (2020)**

**https://doi.org/10.1186/s13063-020-4156-z**

After publication of our article [1] the authors have notified us of inconsistencies between figures and figure legends: Figure 1: the legend is correct; the figure should be current Figure 2. Figure 2: the legend is correct; the figure should be current Figure 3. Figure 3: the legend is correct; the figure should be current Figure 1. Correct Figures and Legends are presented below:

**Fig. 1:**
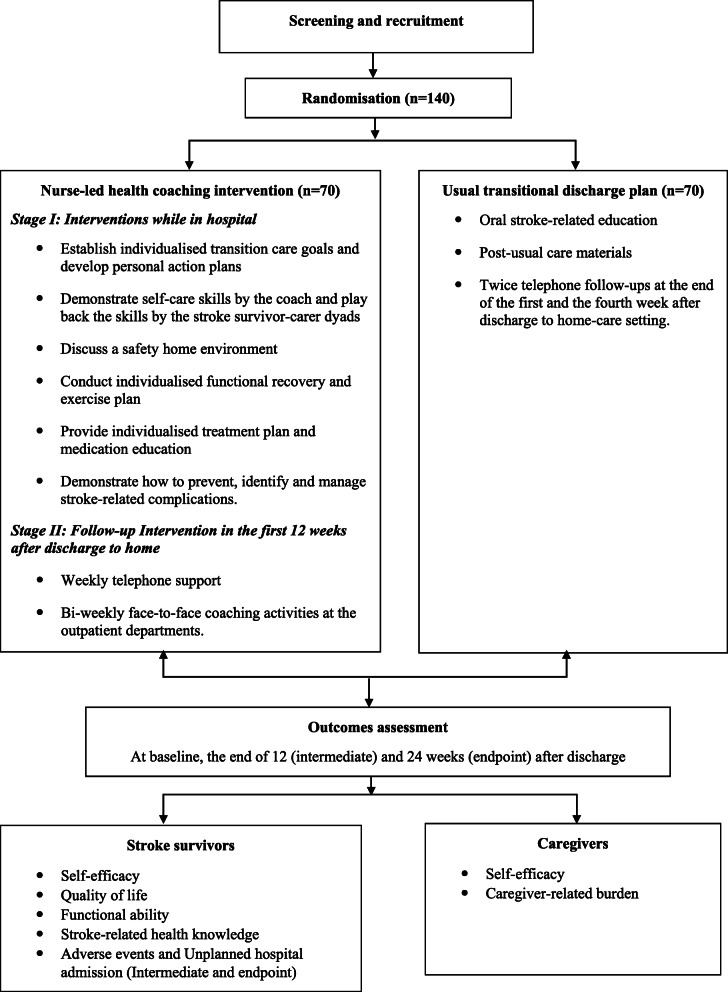
Flow chart of study design

**Fig. 2:**
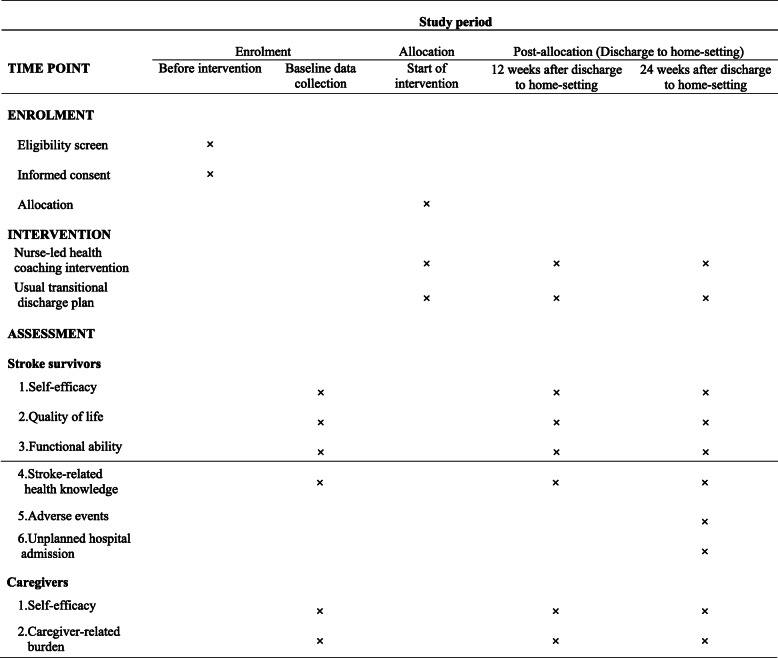
SPIRIT figure: general procedure of enrolment, intervention and assessment

**Fig. 3:**
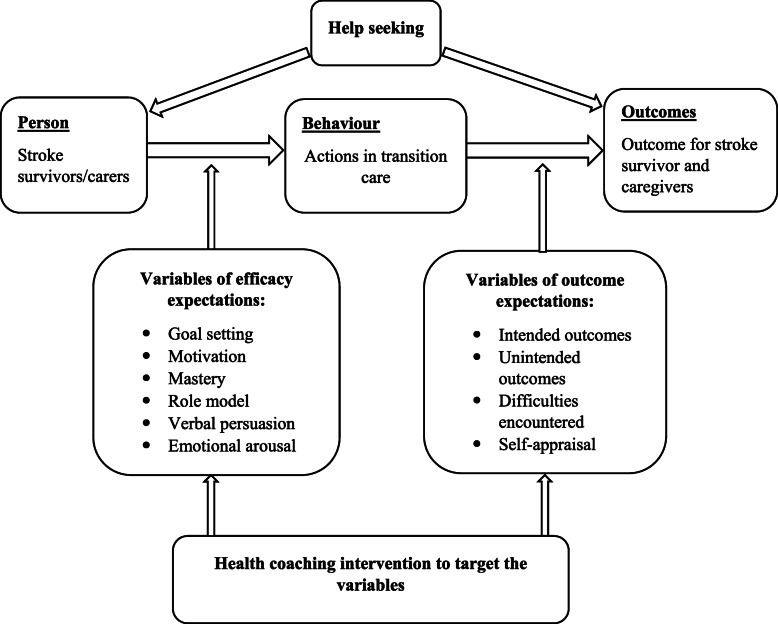
Theoretical framework of the stroke health-coaching program (Adapted from self-efficacy theory)
