# Supplementary material for: A nurse-led health coaching intervention for stroke survivors and their family caregivers in hospital to home transition care in Chongqing, China: a study protocol for a randomized controlled trial
Source: Trials. 2020 Mar 4;21:240. doi: 10.1186/s13063-020-4156-z (PMC7057579; doi:10.1186/s13063-020-4156-z)
Supplement: Supplementary file 2 — Additional file 2. Stroke Health Coaching Diary. [file 13063_2020_4156_MOESM2_ESM.docx]

#### **Additional file 2: Stroke Health Coaching Diary** (Please tick ✓in the spaces of the items you have done prior to each visit)

Code of stroke survivors:

| **Transition goal** | | **Self-management record after discharge** | | | | | |
| --- | --- | --- | --- | --- | --- | --- | --- |
| **Contents** | | **1^st^ month**  **D M Y** | **2^nd^ month**  **D M Y** | **3^rd^ month**  **D M Y** | **4^th^ month**  **D M Y** | **5^th^ month**  **D M Y** | **6^th^ month**  **D M Y** |
| **Self-care skills** | Personal hygiene | Completed ☐  If uncompleted,  times of non-adherence to goals |  |  |  |  |  |
|  | Dressing | Completed ☐  If uncompleted,  times of non-adherence to goals |  |  |  |  |  |
|  | Eating | Completed ☐  If uncompleted,  times of non-adherence to goals |  |  |  |  |  |
|  | Maintaining continence | Completed ☐  If uncompleted,  times of non-adherence to goals |  |  |  |  |  |
|  | Transferring | Completed ☐  If uncompleted,  times of non- adherence to goals |  |  |  |  |  |
| **Functional recovery plan** | __times/day |  |  |  |  |  |  |
| **Medication adherence** | Missed any medication intake | Yes ☐  No ☐ |  |  |  |  |  |
|  | Times of non-adherence medication per month |  |  |  |  |  |  |
|  | Reasons of medication nonadherence |  |  |  |  |  |  |
| **Home environment maintenance** | Lighting in the living area | Yes ☐  No ☐ |  |  |  |  |  |
|  | Floor condition | Yes ☐  No ☐ |  |  |  |  |  |
|  | Handrails in the living area | Yes ☐  No ☐ |  |  |  |  |  |
| **Complications** | Secondary stroke ☐  Falls ☐  Bedsore ☐  Urinary tract infection ☐ |  |  |  |  |  |  |
|  | The numbers of unplanned hospital admission |  |  |  |  |  |  |
