# Supplementary material for: A nurse-led health coaching intervention for stroke survivors and their family caregivers in hospital to home transition care in Chongqing, China: a study protocol for a randomized controlled trial
Source: Trials. 2020 Mar 4;21:240. doi: 10.1186/s13063-020-4156-z (PMC7057579; doi:10.1186/s13063-020-4156-z)
Supplement: Supplementary file 3 — Additional file 3. The model of consent form. [file 13063_2020_4156_MOESM3_ESM.pdf]

Consent Form
(Stroke survivors Version)

Interventional Study - *Adult providing own consent*

Title: A nurse-led health coaching intervention for stroke survivors and their family caregivers in hospital to home transition care in Chongqing

Coordinating Principal Investigator/ Principal Investigator:
A/professor Lily Xiao
College of Nursing and Health Science, Flinders University
Email: lily.xiao@flinders.edu.au

Associate Investigator(s):
A/Professor Diane Chamberlain
College of Nursing and Health Science, Flinders University
Email: di.chamberlain@flinders.edu.au

PhD Candidate Shuanglan Lin
College of Nursing and Health Science, Flinders University
Email: lin0286@flinders.edu.au

Professor Yanjiang Wang
The Third Affiliated Hospital of Army Medical University, Chongqing, China
Email: wayaja@163.com

Associate Professor Zhenfang Chen
The First Affiliated Hospital of Army Medical University, Chongqing, China
Email: chen-zf@hotmail.com

Ms Min Wu
The First Affiliated Hospital of Army Medical University, Chongqing, China
Email: Benben80925@126.com

Ms Lu Mao
The First Affiliated Hospital of Army Medical University, Chongqing, China
Email: maolu200517@163.com

Ms Yingying Shen
The Third Affiliated Hospital of Army Medical University, Chongqing, China
Email: 123473534@qq.com

Ms Anyu Shi
The Third Affiliated Hospital of Army Medical University, Chongqing, China
Email: 18225218261@163.com

Location: The First Affiliated Hospital of Army Medical University, Chongqing, China
The Third Affiliated Hospital of Army Medical University, Chongqing, China

Declaration by Participant

I have read the Participant Information Sheet or someone has read it to me in a language that I understand.

I understand the purposes, procedures and risks of the research described in the project.

I give permission for my intervention nurses to have access and release my medical record information and feedbacks from questionnaires with other researchers in this project, to collect, analyse, publish and report in journals and thesis. I understand this is only in the First Affiliated Hospital of Army Medical University, and the Third Affiliated Hospital of Army Medical University Chongqing, China concerning my disease and treatment for the purposes of this project. I understand that such information will remain confidential.

I understand this research forms part of the hospital's performance in research and staff development requirements.

I have had an opportunity to ask questions and I am satisfied with the answers I have received.

I freely agree to participate in this research project as described and understand that I am free to withdraw at any time during the study without affecting my future health care.

I understand that I will be given a signed copy of this document to keep.

Name of Participant (please print) ______________________________

Signature ______________________ Date ______________________

Name of Witness* to Participant's Signature (please print) ______________________________

Signature ______________________ Date ______________________

* Witness is not to be the investigator, a member of the study team or their delegate. In the event that an interpreter is used, the interpreter may not act as a witness to the consent process. Witness must be 18 years or older.

Declaration by Senior Researcher†

I have given a verbal explanation of the research project, its procedures and risks and I believe that the participant has understood that explanation.

Name of Study Doctor/
Senior Researcher† (please print) ______________________________

Signature ______________________ Date ______________________

† A senior member of the research team must provide the explanation of, and information concerning, the research project.

Note: All parties signing the consent section must date their own signature.

Consent Form - *Adult providing own consent*
(Family Caregivers Version)

Title: A nurse-led health coaching intervention for stroke survivors and their family caregivers in hospital to home transition care in Chongqing

Coordinating Principal Investigator/ Principal Investigator:
A/professor Lily Xiao
College of Nursing and Health Science, Flinders University
Email: lily.xiao@flinders.edu.au

Associate Investigator(s):
A/Professor Diane Chamberlain
College of Nursing and Health Science, Flinders University
Email: di.chamberlain@flinders.edu.au

PhD Candidate Shuanglan Lin
College of Nursing and Health Science, Flinders University
Email: lin0286@flinders.edu.au

Professor Yanjiang Wang
The Third Affiliated Hospital of Army Medical University, Chongqing, China
Email: wayaja@163.com

Associate Professor Zhenfang Chen
The First Affiliated Hospital of Army Medical University, Chongqing, China
Email: chen-zf@hotmail.com

Ms Min Wu
The First Affiliated Hospital of Army Medical University, Chongqing, China
Email: Benben80925@126.com

Ms Lu Mao
The First Affiliated Hospital of Army Medical University, Chongqing, China
Email: maolu200517@163.com

Ms Yingying Shen
The Third Affiliated Hospital of Army Medical University, Chongqing, China
Email: 123473534@qq.com

Ms Anyu Shi
The Third Affiliated Hospital of Army Medical University, Chongqing, China
Email: 18225218261@163.com

Location: The First Affiliated Hospital of Army Medical University, Chongqing, China
The Third Affiliated Hospital of Army Medical University, Chongqing, China

Declaration by Participant

I have read the Participant Information Sheet or someone has read it to me in a language that I understand.

I understand the purposes, procedures and risks of the research described in the project.

I understand this research forms part of the hospital's performance in research and staff development requirements.

I have had an opportunity to ask questions and I am satisfied with the answers I have received.

I freely agree to participate in this research project as described and understand that I am free to withdraw at any time during the study.

I understand that I will be given a signed copy of this document to keep.

Name of Participant (please print) ______________________________	
Signature ______________________	Date ______________________

Name of Witness* to Participant's Signature (please print) ______________________________	
Signature ______________________	Date ______________________

* Witness is not to be the investigator, a member of the study team or their delegate. In the event that an interpreter is used, the interpreter may not act as a witness to the consent process. Witness must be 18 years or older.

Declaration by Senior Researcher†

I have given a verbal explanation of the research project, its procedures and risks and I believe that the participant has understood that explanation.

Name of Study Doctor/ Senior Researcher† (please print) ______________________________	
Signature ______________________	Date ______________________

† A senior member of the research team must provide the explanation of, and information concerning, the research project.

Note: All parties signing the consent section must date their own signature.
